# Noninvasive Trans-Spinal Direct Current Stimulation Improves Breathing in Mice Modeling an Obesity-Associated Central Hypoventilation Syndrome

**DOI:** 10.1007/s12264-026-01680-9

**Published:** 2026-07-24

**Authors:** Roman Delucenay-Clarke, Alexis Casciato, Marlène Bret, Manon Reverdy, Alain Frugière, Frédéric Blond, Noémie Bonneau, Elodie Reboussin, Annabelle Réaux-Le Goazigo, Xavier Guillonneau, Jean-Charles Lamy, Thomas Similowski, Marie-Cécile Niérat, Florence Cayetanot, Laurence Bodineau

**Affiliations:** 1 Inserm, UMR_S1158 Neurophysiologie Respiratoire Expérimentale et Clinique, Sorbonne Université, 75013 Paris, France; 2 CNRS, Inserm, Institut de la Vision, Sorbonne Université, 75012 Paris, France; 3 Institut du Cerveau, Inserm UMR_S1127, CNRS UMR 7225, Sorbonne Université, 75013 Paris, France; 4 Centre de NeuroImagerie de Recherche, Institut du Cerveau, Hôpital Pitié-Salpêtrière, 75013 Paris, France; 5 Département R3S (Respiration, Réanimation, Réadaptation respiratoire, Sommeil), AP-HP, Groupe Hospitalier Universitaire APHP-Sorbonne Université, Hôpital Pitié-Salpêtrière, 75013 Paris, France

## Dear Editor,

Obesity Hypoventilation Syndrome (OHS) is a neurorespiratory disorder associated with obesity, affecting an estimated 0.4% of the world's adult population, and for which there is no effective curative treatment. OHS reduces quality of life by causing notable cardiorespiratory complications, leading to a higher risk of mortality. Its prevalence reaches 11% for patients with a body mass index (BMI) of 30–35 kg/m^2^, 20% for a BMI of 35–40, 30% for a BMI of >40, and up to 48% for a BMI > 50 kg/m^2^ [1]. OHS is conventionally defined as the combination of obesity (BMI >30 kg/m^2^), daytime hypercapnia (awake arterial pCO_2_ >45 Torr), and sleep-disordered breathing without an alternate explanation for hypoventilation (such as restrictive lung or chest wall disorders, use of depressant drugs, or other central hypoventilation syndrome) [1]. In addition to the mechanical load induced by obesity, alterations in central respiratory drive are recognized as having a determining role in the occurrence of OHS, which seems to be an acquired phenomenon that involves central leptin resistance [1].

We previously reported that upper cervical cathodal trans-spinal direct current stimulation (tsDCS) in heathy human led to an increase in cortico-diaphragmatic excitability and tidal volume (V_T_) during a single session, combined with an increase in respiratory frequency (f_R_) during repeated sessions. These effects occurred both during stimulation and after its cessation [2–4]. The underlying mechanisms may involve transient changes in membrane potential, synaptic changes via long-term potentiation/depression-like processes, or non-synaptic effects involving neurogenesis, all of which have been described as underlying the neuroplasticity observed with DCS [3, 5, 6]. Whatever the mechanism, cathodal tsDCS applied to the upper cervical region could provide an avenue for therapeutic studies evaluating tsDCS as a tool to increase breathing in patients with central hypoventilation, such as those with OHS.

**Fig. 1:**
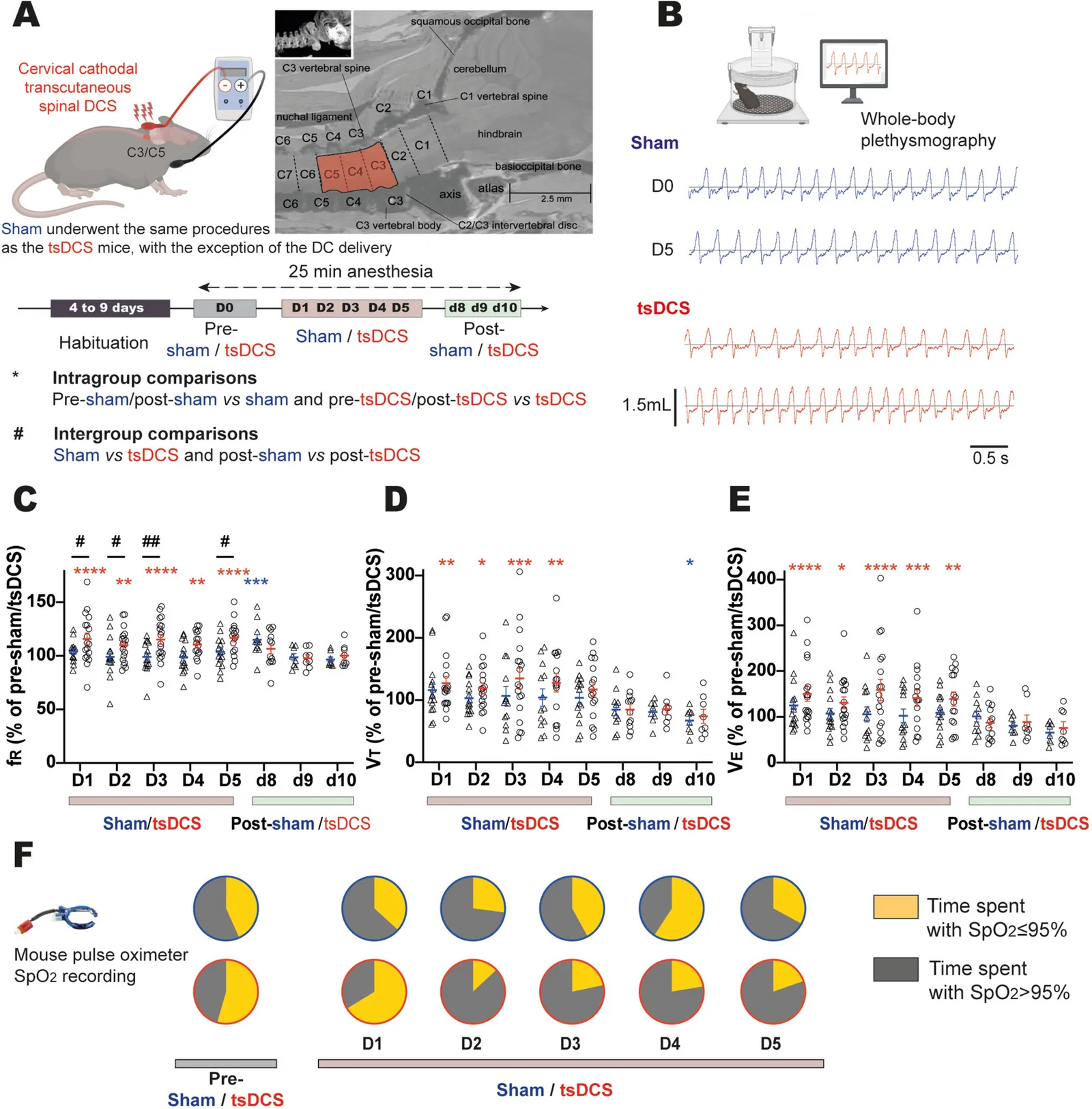
Breathing in *Lep*^*ob/ob*^ mice modeling OHS following cathodal tsDCS targeting the upper cervical region of the spinal cord. **A** Placement of electrodes targeting the C3/C5 spinal cervical region and time lag to study the effect of tsDCS. MRI image; License number 6123621428706 [18]. Adapted from “Mouse (lateral1, curved tail)”, “Mouse CNS (lateral, to coccygeal nerves)” and “tDCS device”, by BioRender.com 2022; retrieved from https://app.biorender.com/biorender-templates. **B** Individual plethysmograph traces. **C**–**E** Individual values with mean ± SEM superimposed (sham, triangles, *n =*15; tsDCS, circles, *n =*18) of the respiratory frequency (f_R_; **C**), tidal volume (V_T_; **D**), and minute ventilation ($\dot{\mathrm{V}}_{\mathrm{E}}$; **E**). *, intragroup comparisons (repeated measure ANOVA or mixed model followed by the Benjamini, Krieger, and Yekutieli multiple comparison test; f_R_ *F=*6.531, *P*<0.0001; V_T_ *F=*6.597, *P*<0.0001; $\dot{\mathrm{V}}_{\mathrm{E}}$ *F=*6.310, *P*<0.0001) and #, intergroup comparisons (ANOVA two-way or mixed model followed by the Benjamini, Krieger, and Yekutieli multiple comparison test; f_R_ *F*=3.594, *P*<0.0006); * *P*<0.05, ** *P*<0.01, *** *P*<0.001, **** *P*<0,0001, # *P*<0.05, ## *P*<0.01. **F** Pie chart showing the average time mice spent with SpO_2_≤95% (hypoventilation) and with SpO_2_>95%.

Since cathodal cervical tsDCS stimulates ventilation by a central effect in healthy humans, and assuming that this effect is based on neuroplasticity, we postulated that cathodal tsDCS applied to the upper cervical region in leptin-deficient obese mice (*Lep*^*ob/ob*^), mimicking human OHS (increased PaCO₂ and reduced ventilatory response to CO_2_) [7], would improve ventilation by modulating the activity of neurons in the neural network that controls breathing. To validate our hypothesis, we first transposed the non-invasive transcutaneous cervical tsDCS approach used in humans to adult mice [2, 4] (Fig. 1A) and then combined whole-body plethysmographic recording, SpO_2_ measurement, functional and phenotypic histology, and RNA sequencing analysis. Since our protocol consisted of 5 consecutive days of cervical tsDCS (Fig. 1A), only male mice were used in order to avoid the fluctuations in sex hormones that are inevitable in females over 5 consecutive days due to the duration of their ovarian cycle, which prevents the formation of a homogeneous group of females synchronized to a single phase throughout the duration of the tsDCS protocol.

Overall, tsDCS significantly enhanced the ventilation (V̇_E_) of *Lep*^*ob/ob*^ mice by increasing f_R_ and V_T_ (Fig. 1B, 1C, 1D, 1E). Comparing tsDCS values with pre-tsDCS showed that f_R_ was increased by +16% from the first day of stimulation (D1) and remained increased until D5 (Fig. 1C). The comparison between tsDCS and sham groups indicated that tsDCS mice had a significantly higher f_R_ than sham mice every day except on D4 (Fig. 1C). V_T_ was increased in tsDCS mice from D1 to D4 in comparison with pre-tsDCS (Fig. 1D). Finally, and aligned with the above, there was an increase from pre-tsDCS in V̇_E_ during the whole tsDCS procedures (Fig. 1E). It is important to note, however, that repeated anesthesia may have contributed to moderating the stimulating effect of tsDCS due to a potential depressant effect on ventilation. Also, cathodal cervical tsDCS improved *Lep*^*ob/ob*^ deoxygenation by reducing their time spent in hypoventilation (SpO_2_ ≤95%). Although SpO_2_ did not increase significantly throughout the duration of the recording sessions (95.4% [94.7;95.9] at D0 and 95.4% [92.1;96.9] at D5), tsDCS mice spent less time hypoventilating than sham mice (Fig. 1F; Table S1); they had a significant reduction in time spent with SpO_2_ ≤95% compared to pre-tsDCS from D2 to D5. Intergroup comparison revealed an increase in the time spent with SpO_2_ ≤95% at D1 in tsDCS compared to the sham mice (*P*=0.0354), and conversely, an increase in this duration at D4 in sham compared to tsDCS mice (Fig. 1F; Table S1). Note that cathodal cervical tsDCS also increases the V̇_E_ of healthy mice (Table S2). The ventilatory effect of tsDCS appeared to be specific, as we observed no changes in other physiological variables (see supplements).

**Fig. 2:**
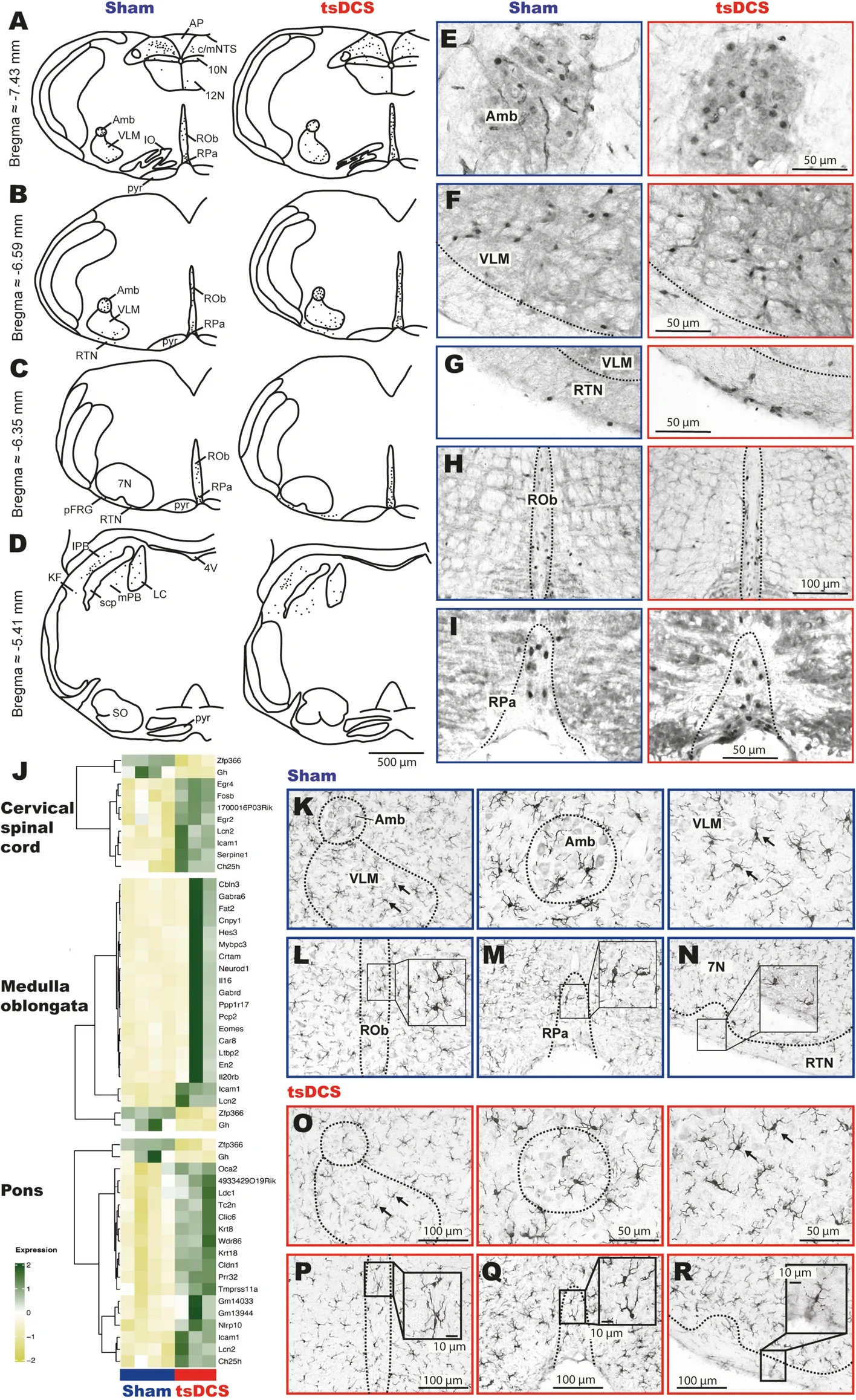
Changes in *c-Fos* expression within the brainstem respiratory network concomitant with a phenomenon of neuroplasticity without neuroinflammatory processes in *Lep*^*ob/ob*^ mice submitted to cervical cathodal tsDCS. **A**–**I** Distribution of cFOS-positive cells at four rostro-caudal levels of the medulla oblongata and pons. **J** Heatmaps illustrating RNA-seq differential expression data in the cervical spinal cord, medulla oblongata, and pons (**F**). Green, positive log fold-change indicates higher expression in the tsDCS tissue compared with the sham tissue; yellow, negative log FC. **K**–**R** Photomicrographs illustrating the morphology of microglial cells in respiratory-related structures presenting a change in cFOS distribution. 10N, dorsal motor nucleus of the vagus; 12N, hypoglossal nucleus; 4V, 4^th^ ventricle; 7n, facial nerve; 7N, facial nucleus; Amb, ambiguus nucleus; AP, area postrema; c/mNTS, commissural and median parts of the nucleus of the tractus solitarius; IO, inferior olive; KF, Kölliker fuse nucleus; LC, locus coeruleus; lPB, lateral subdivision of the parabrachial nucleus; mPB, median subdivision of the parabrachial nucleus; pFRG, parafacial respiratory group nucleus; pyr, pyramidal tract; ROb, raphe obscurus nucleus; RPa, raphe pallidus nucleus; RTN, retrotrapezoid nucleus; scp, superior cerebellar peduncle; SO, superior olive; VLM, ventrolateral medullary reticular nucleus.

To assess the effect of cervical cathodal tsDCS on the brainstem respiratory neural network in *Lep*^*ob/ob*^ mice, we compared *cFos* expression between tsDCS and sham mice in the medulla oblongata and pons, *i.e.,* brainstem regions that contain the respiratory network controlling the central respiratory drive delivered to thoracoabdominal respiratory muscles [8]. In the medulla oblongata, we observed a significant increase in ventral structures: ambiguous nucleus (Amb; Fig. 2A-2B and 2E, Table S3), ventrolateral medullary reticular nucleus (VLM; Fig. 2A- 2B and 2F, Table S3), Raphe Pallidus (RPa; Fig. 2A-2C and 2I, Table S3), Raphe Obscurus (ROb; Fig. 2A-2C and 2H, Table S3), and in the retrotrapezoid nucleus (RTN; Fig. 2C and 2G, Table S3). In the pons, the number of cFOS-positive cells did not significantly vary in the analyzed respiratory-related structures (Fig. 2D, Table S3).

To investigate the potential mechanisms by which cervical cathodal tsDCS could stimulate spontaneous breathing and change *cFos* expression in *Lep*^*ob/ob*^ mice, we conducted RNAseq analysis focused on the cervical spinal cord, where tsDCS had been applied, as well as on the medulla oblongata and pons. This study was exploratory in nature due to the small number of samples analyzed, but it suggested findings consistent with the literature on tsDCS/DCS. Only a small number of genes had their expression modulated by tsDCS, with a significant proportion of them associated with a phenomenon of plasticity (Fig. 2J; Table S4): *Egr2*, *Egr4* and *Fosb* encoding transcription factors associated with neuroplasticity; *Car8*, *Gabra6*, *Gabrad* and *Clic6* directly associated with neurotransmission; *Cbln3* and *Gh* involved in maintaining neuronal integrity or synapse structure and *En2* involved in the stabilization or development of neural circuits. The regulation of genes encoding transcription factors associated with this neuroplasticity was corroborated by RT-qPCR: in cervical spinal cord, *Egr2* (mRNA fold induction sham, 0.8350 [0.6325;2.088]; tsDCS 3.525 [2.1733;5.478]; *P*<0.04), *Egr4* (sham, 1.010 [0.6425;1.610]; tsDCS 6.895 [4.190;7.320]; *P*<0.002) and *Fosb* (sham, 0.8250 [0.7275;1.1770]; tsDCS 5.620 [4.165;6.565]; *P*<0.03) and in medulla oblongata *Egr4* (sham, 1.055 [0.7125;1.360]; tsDCS 1.735 [1.433;1.948]; *P*<0.03) and *Fosb* (sham, 1.065 [0.7450;1.288]; tsDCS 1.740 [1.430;1.840]; *P*<0.02).

Finally, to assess whether or not the respiratory effects of tsDCS involved modulation of neuroinflammatory processes, we carried out a morphological analysis of microglia in structures showing tsDCS-induced changes in the density of cFOS-positive neurons. We did not observe any changes in microglial cell density or in the number and length of cell branches in any structure related to respiratory control that showed a change in cFOS distribution (Table S5). These data are illustrated by photomicrographs in the Amb (Fig. 2K and 2O), ROb (Fig. 2L and 2P), RPa (Fig. 2M and 2Q), RTN (Fig. 2N and 2R), and VLM (Fig. 2K and 2O). Data obtained by RNAseq analysis showed that very few genes involved in inflammatory processes were modulated (Table S4): *Icam1* and *Lcn2* in the three analyzed regions, *Il16, Il20rb, Crtam* in the medulla oblongata, and *Nlrp10* in the pons.

Collectively, our study established a breathing-stimulating effect of transcutaneous cathodal tsDCS at the level of the upper cervical region in a preclinical model of central hypoventilation syndrome, the *Lep*^*ob/ob*^ mice. This stimulating effect led to a reduction of hypoventilation and a stimulation of neuronal activity in the respiratory brainstem network concurrently with a phenomenon of neuroplasticity. Note that this effect is also present in healthy WT mice, a finding consistent with the data we obtained in healthy subjects [2] that support the idea of a respiratory stimulant effect regardless of whether or not central hypoventilation was present. While many works and clinical trials provided solid evidence for the use of transcranial DCS in neurological disorders such as stroke or movement disorders or in learning [9], our data suggest for the first time that tsDCS applied at the upper cervical level can correct central defects in respiratory control. The stimulation of breathing induced by cervical cathodal tsDCS depended on an effect sustained by an increase in both f_R_ and V_T_—with a greater effect on f_R_— which was sufficiently effective to reduce the time spent in hypoventilation. It should be noted, however, that the average SpO_2_ of the mice was unchanged. We assume this is related to the fact that the animals were awake during recording, which minimizes hypoventilation. We observed an increase in f_R_ from the first stimulation session, whereas in healthy humans, this was not observed until after repeated stimulation [4]. Such an effect in f_R_ suggests that cervical cathodal tsDCS leads to modulation of the ponto-medullary respiratory network, and especially of the respiratory rhythm generators in the medulla oblongata [8]. Previous modelling of the electric field generated by the application of tsDCS in C3-C5 in humans has shown longitudinal invasion of the brainstem. It may be due to the cable-structure of the spine with a conductive core (spinal cord and cerebrospinal fluid) surrounded by an insulating sheath (vertebral column) [2, 4, 10]. In the work of Fernandes and collaborators [10], Fig. C of their supplementary information indicates that the ventral and dorsal parts of the medulla oblongata and the dorsal part of the pons should be affected by the electric field generated by tsDCS applied at C3-C5; data fully compatible with modulation of the ponto-medullary respiratory network [8]. Another possibility is that the stimulation of the ponto-medullary respiratory structures depends on the modulation of ascending sensitive pathways. In support of this possibility, it has previously been reported that cathodal tsDCS tends to increase the amplitude of somatosensory potentials evoked by peripheral nerve stimulation [11]. In both cases, it appears that in mice as in humans, cervical tsDCS affects the respiratory neuronal network located in the brainstem. Our cFOS data suggested that the brainstem respiratory structures affected are limited to those located in the ventral portion of the medulla oblongata, a feature fully compatible with a diffusion of the electric field to this region as assumed in humans [10]. A greater number of cFOS-positive neurons in the VLM of tsDCS mice suggests an increase in activity in the ventral respiratory group, a functional entity that includes the pre-Bötzinger complex, rhythmic generator of inspiration [3, 8]. Our data also suggest that cervical cathodal tsDCS led to enhanced neuronal activity in neuronal groups involved in CO_2_/H^+^ chemosensitivity, *i.e.*, the RTN, whose PHOX2B neurons are thought to be the main site of this chemosensitivity [8], and RPa and ROb nuclei, which contain serotonergic acidosis-stimulated neurons [8]. Whether by excitation of the inspiration-generating neurons of the pre-Bötzinger complex or neighboring neurons, or by excitation of neurons involved in stimulating respiratory drive during gaseous challenges, both f_R_ and V_T_ can be increased. It's worth noting that although the analysis was conducted specifically in structures of the respiratory network, we cannot completely rule out the possibility that the changes in *cFos* expression induced by tsDCS are related to other physiological effects. On the other hand, no change in the density of cFOS-positive cells in the c/mNTS was observed. It suggests that cervical cathodal tsDCS did not influence respiratory control by modulating ascending neural pathways. As the c/mNTS contributes to ventilatory homeostasis by directly or indirectly integrating information from sensory receptors such as rib cage mechanoreceptors or carotid bifurcation chemoreceptors [8, 12], an increase in signals from these nerve pathways would have led to a rise in the number of c/mNTS cFOS-positive neurons. Nevertheless, the refutation of the hypothesis that tsDCS exerts a ventilatory effect by modulating ascending sensory pathways is based solely on our cFOS data; this issue, therefore, warrants further investigation. Of course, a direct effect on the phrenic motoneurons or even on the phrenic nerve itself could contribute to the increase in V_T_ that we observed. Regarding an effect on the phrenic motoneurons, it could involve a modulation of calcium conductance, which has been shown to contribute to the effects of cathodal tsDCS on motoneurons [13].

The cellular mechanisms by which weak electric fields induced by DCS produce their effects are not yet fully elucidated. However, it is generally accepted that changes in neuronal resting membrane potential are responsible for the immediate regulatory effects as well as the after-effects that persist for several minutes or hours [5]. These effects that may involve neuroplasticity phenomena or modulation of neuroinflammatory processes. Consistent with the idea of inducing neuroplasticity processes, given that our approach to analyzing variations in gene expression is still exploratory, our RNA-seq and RT-qPCR data suggested a modulation of the expression of immediate early genes encoding transcription factors previously involved in orchestrating gene expression changes that underlie neuronal plasticity, *i.e., Egr2*, *Egr4,* and *Fosb*. Also, our RNAseq data suggested a tsDCS-induced over-expression of genes directly involved in the GABA/GABA_A_ signaling (*Car8*, *Gabra6*, *Gabrad*) in the medulla oblongata. This is an interesting observation, given that GABA/GABA_A_ signaling is one of the signaling pathways involved in the elaboration and regulation of central respiratory drive [8], and that a growing number of animal and human studies in recent years have shown DCS-induced changes in GABAergic signaling [14]. In addition, our data do not support the potential contribution of tsDCS-induced modulation of neuroinflammatory processes to the breathing effects we observed, either through glial cells stimulation and promotion of pro-inflammatory cytokine production as previously demonstrated following DCS [15], or by reducing obesity-related neuroinflammation [16]. The expression of very few genes involved in inflammatory processes was modulated by tsDCS, and the analysis of microglial cell morphology in respiratory-related structures with tsDCS-induced increases in cFOS levels revealed no change [17].

To conclude, our data showed that cervical cathodal tsDCS improved the breathing of leptin-deficient mice modelling OHS. It seems to involve both a stimulating action on respiratory network structures and an induction of neuroplasticity processes. Our findings provide proof of concept for a new non-pharmacological and non-invasive approach to the treatment of hypoventilation in OHS patients. This work opens up prospects for the use of this approach in the more general context of central hypoventilation syndromes. Future studies in female mice, examining other physiological variables, in the potential conditions under which long-term effects may persist, and on cellular and molecular processes, notably by functional validation, are essential to fully assess the translational potential of this approach.

## Supplementary Information

Below is the link to the electronic supplementary material.

Supplementary file1 (PDF 348 KB)

Supplementary file2 (PDF 319 KB)
