# Supplementary material for: Noninvasive Trans-Spinal Direct Current Stimulation Improves Breathing in Mice Modeling an Obesity-Associated Central Hypoventilation Syndrome
Source: Neurosci Bull. 2026 Jul 24;42(10):2393–8. doi: 10.1007/s12264-026-01680-9 (PMC13642236; doi:10.1007/s12264-026-01680-9)
Supplement: Supplementary file 1 [file 12264_2026_1680_MOESM1_ESM.pdf]

Supplementary Materials

Materials and Methods

Experiments were carried out according to Directive 2010/63/EU of the European Parliament and of the French Law Council (22 September 2010; 2013/118). Animal protocols referenced APAFIS#18893-2019013016523974 v4 and APAFIS#26676-2020070910424067 v3 were approved by the Charles Darwin Ethical Committee for Animal Experimentation. All efforts were made to minimize the number of animals and their suffering.

Animals

Experiments were performed on adult male mice: wildtype (WT) Swiss OF1 strain (49.3±1.1g, 13-17 weeks old, n = 22; Charles River Laboratories, L'Arbresle, France) and B6. V *Lep*$^{ob/ob}$ JRj mice (hereafter termed *Lep*$^{ob/ob}$ mice, 49.8±8.7g, 10-25 weeks old, n = 62; Janvier labs, Le Genest-Saint-Isle, France). *Lep*$^{ob/ob}$ mice were chosen as a model for obesity hypoventilation syndrome (OHS) because they display an elevated $PaCO_2$ level indicative of chronic hypercapnia and a reduced ventilatory response to CO_2, as OHS patients [1, 2]. All mice were maintained under controlled temperature and humidity (22°C ±1°C and 33%, respectively) with a 12h light-dark cycle. Food and water were provided *ad libitum*.

Cathodal tsDCS Procedures in the Upper Cervical Region

Mice were randomly distributed into 2 groups, tsDCS (OF1 n = 11; *Lep*$^{ob/ob}$ n = 32) or sham (OF1 n = 11; *Lep*$^{ob/ob}$ n = 30). Cathodal tsDCS was performed on anesthetized mice. Anesthesia was initiated with 3% isoflurane (ISO-VET, Laboratoire OSALIA, Paris, France) and then maintained with coaxial mask at 2.5% isoflurane, and mice were placed, backside towards the experimenter, on heating pads (Equipement vétérinaire Minerve ®, Esterney, France). Electrical stimulation was made using disposable surface electrodes (NeuroTab – spesmedica, Genoa, Italy), modified by cutting into a 7x7mm square shape and connected to a direct current stimulator (NeuroConn_DC-Stimulator, NeuroCare Group, Munich, Germany). The negative current output from the stimulator was connected to an electrode placed on the dorsal region of the neck, targeting the C3/C5 cervical segments of the spinal cord (cathode electrode; Fig. 1A). The cathode electrode placement was based on palpation of the C2 vertebral spine positioned just caudal to the rostral end of the C3 cervical segment (Fig. 1A). The anode electrode was placed ventrally opposite the cathode. Cathode and anode were applied to shaved skin that was covered with electrically conductive gel (Asept inmed, Quint Fonsegrives, France). Stimulus intensity was set at 2 mA and was applied over a 20 min period [3], resulting in a current density of 4.08 mA/cm^2 and a delivered total charge of 4.9 C/cm^2. The

current was ramped up to 2 mA over a 10 s period and similarly ramped down at the end of stimulation. Sham mice underwent the same procedures as the tsDCS group, with the exception of the application of electrical stimulation. Following the 20 minutes of electrical stimulation (or without stimulation for the sham mice), anesthesia was lifted for both tsDCS and sham mice (about 25 minutes of anesthesia for both groups [duration included time taken for all mice to be fully anesthetized, the positioning of electrodes, and the electrical stimulation if applicable]). For OF1 as for $Lep^{ob/ob}$ mice, tsDCS or sham procedures were performed for five consecutive days by the same researcher at the same time every day.

Recording of Ventilatory Variables

Breathing variables were recorded in tsDCS (OF1, n = 11; $Lep^{ob/ob}$ n = 18) and sham (OF1, n = 11; $Lep^{ob/ob}$ n = 15) mice by whole-body plethysmography under normocapnia. The recording protocol was carried out over 3 weeks (Fig. 1A). In the first week, mice were acclimatized to plethysmograph chambers (DSI Buxco® Multi-function Bias Flow, Harvard Bioscience, Holliston, USA, or Emka Technologies, Paris, France) for 1 hour on 4 consecutive days. On the 5th day, pre-tsDCS/sham values were obtained by recording the breathing (DSI data acquisition system, Ponemah® Software v5.40, Harvard Bioscience, Holliston, USA or Emka Technologies, IOX version 2.10.5.14, Paris, France) after mice were anesthetized for 25 minutes and after 20 minutes of fully waking up from the anesthesia (D0; Fig. 1A). In the second week, on 5 consecutive days, mice were subjected to tsDCS or sham procedures and their breathing was recorded in the same conditions as on D0 (D1-5; Fig. 1A). In the third week, breathing was recorded in the same conditions as on D0 without electrical stimulation (d8-10, post-tsDCS/sham; Fig. 1A). In periods without body movement, plots were analyzed in order to evaluate the respiratory frequency (f_R, respiratory cycles. min^{-1}), tidal volume (V_T, µL) normalized as the ratio of V_T divided by body weight (V_T, µL.g^{-1}) and minute ventilation ($\dot{V}_E$, mL.g^{-1}.min^{-1}). Body weight was measured concurrently with the respiratory variables.

SpO_2 Measurement and Heart Rate

SpO_2 measurements and heart rate were performed on awake $Lep^{ob/ob}$ mice (tsDCS, n = 5, and sham, n = 5; Fig. 1F) using a pulse oximeter (MouseOx® Plus, StarrLife Sciences Corp., Oakmont, USA) following a protocol conducted over 3 weeks. In the first week, mice were accustomed to SpO_2 neck sensors for 5-10 minutes for 4 days, and with cabled mouse collar sensors on the 5th day. In the second week, the acclimatation with cabled mouse collar sensors resumed for 4 days and on the 5th day, pre-tsDCS/sham values were obtained after mice had been anesthetized for 25 minutes and after the total lifting of the anesthesia (D0; Fig. 1F). In the third week, on 5 consecutive days, mice were subject to either tsDCS or sham procedures and their SpO_2 and heart rate was recorded under the same conditions as on D0 (D1-5; Fig. 1F).

Tissue Collection

Lep$^{ob/ob}$ mice tissues were collected after tsDCS or sham procedures, and stored either after tissue fixation for an immunohistological analysis (tsDCS, n = 6 and sham, n = 6), or directly by freezing at −80°C for RNA sequencing analysis (tsDCS, n = 3 and sham, n = 4). Tissue fixation was done by transcardially perfusing deeply anesthetized mice (100 mg/kg ketamine and 10 mg/kg xylazine cocktail to which was added 0.1 mg/kg of the analgesic buprenorphine) with a 4% solution of paraformaldehyde (PFA) in 0.1 mol/L phosphate-buffered saline (PBS). Brains were collected, post-fixed for over 48 hours at 4°C with 4% PFA solution, and then conserved with 0.1% sodium azide PBS until histological processing. Non-fixed tissues were collected immediately after deep anesthesia (100 mg/kg ketamine and 10mg/kg xylazine cocktail to which was added 0.1 mg/kg of the analgesic buprenorphine). The central nervous system was harvested *en bloc* while the animals were kept at 4°C and immediately micro-dissected on ice to isolate the cervical spinal cord, medulla oblongata, and pons. The cervical spinal cord and the two encephalic subdivisions were stored directly at -80°C.

Immunohistochemistry

Brainstems were cryoprotected for 48 hours in 0.1 mol/L phosphate buffer containing 30% sucrose, and free-floating coronal sections (30 µm) were obtained using a cryostat (Leica CM 1510S). Sections were divided into 2 sets (one section out of two). One set was used for the detection of the neuronal activity marker cFOS to identify the respiratory-related structures modulated by tsDCS. The second set was used for the detection of the microglia-specific protein ionized calcium binding adaptor molecule 1 (Iba1) with the aim of evaluating the microglial morphology to measure the neuroinflammation. For both sets, sections from the medulla oblongata and pons from tsDCS and sham mice were processed in parallel.

For cFOS co-detection, sections were first incubated with a cFOS antibody (1:4000; sc-253; Santa Cruz Biotechnology Inc., Santa Cruz, CA, USA) in 1% BSA (48h, 4°C). Then, they were incubated with a biotinylated goat anti-rabbit IgG antibody (BA-1000, Vector Laboratories, Burlington, Canada; 1:500; in 1% BSA; 2 h, room temperature), followed by an avidin-biotin-peroxidase complex (ABC kit standard, PK-6100, Vectastain, Vector Laboratories, Burlingame, CA, USA; 1:250; 1h). Peroxidase activity was detected using a solution containing 0.02% 3,3'-diaminobenzidine tetrahydrochloride, 0.04% nickel ammonium sulfate and 0.01 % H_2O_2 in 0.05 M Tris-HCl buffer (pH 7.6), which resulted in a blue/grey color in the nucleus. Some sections were processed in parallel, but with the omission of primary or secondary antibodies; no labelling was observed in these conditions. All sections were mounted in sequential caudo-rostral order on silanized slides, air-dried, and coverslipped with EUKITT (Bio Optica, Milan, Italy). cFOS positive

cells were counted in structures involved in elaboration or adaptation of the respiratory drive localized using standard landmarks [4-6]: in the commissural and medial parts of the nucleus of the solitary tract (c/mNTS), in the hypoglossal nucleus (12N), in the dorsal motor of the vagus (10N), in the ventrolateral medullary reticular nucleus (VLM; a neuronal column ventral to nucleus ambiguous, extending from pyramidal decussation to caudal edge of facial nucleus, that contains the ventral respiratory column), in the medullary raphe nuclei *i.e.* raphe obscurus (ROb) and raphe pallidus (RPa), in the perifacial ventral medullary surface at the level of the region containing the retrotrapezoid nucleus (RTN) and parafacial respiratory group (pFRG) [7-9], in the locus coeruleus (LC), in the lateral and medial parts of the parabrachial nucleus (lPB and mPB), and in the Kölliker-Fuse nucleus (KF). cFOS immunolabeled cells were visually counted by an investigator blinded to samples using a light microscope (Leica DM2000; Leica Microsystems, Heidelberg, Germany) at high magnification (x200 or x400, depending on the immunolabeled density of cells). Immunolabelling for cFOS was photographed with a digital camera (Leica DFC450C, Leica Microsystems, Heidelberg, Germany) using Leica Application Suite software (L.A.S V4.5).

For Iba1 detection, sections were incubated with an Iba1 antibody (1:1000; 019-19741; Fujifilm, FujiFilm Wako, Chuo-Ku, Osaka, Japan) in 1% BSA (48h, 4°C). Then they were incubated with a biotinylated horse anti-rabbit IgG antibody (BA-1100, Vector Laboratories, Burlington, Canada; 1:500; in 1% BSA; 2 h, room temperature), followed by an avidin-biotin-peroxidase complex (ABC kit standard, PK-6100, Vectastain, Vector Laboratories, Burlingame, CA, USA; 1:250; 1 h). Peroxidase activity was detected using a solution containing 0.02% 3,3'-diaminobenzidine tetrahydrochloride, 0.04% nickel ammonium sulfate, and 0.01 % H_2O_2 in 0.05 mol/L Tris-HCl buffer (pH 7.6). Some sections were processed in parallel, but with the omission of primary or secondary antibodies; no labelling was observed in these conditions. All sections were mounted in sequential caudo-rostral order on silanized slides, air-dried, and coverslipped with EUKITT (Bio Optica, Milan, Italy). Iba1-positive cells were analyzed in the respiratory-related structures, showing a difference in cFOS-positive neuron density between the tsDSC and sham conditions from microphotographs obtained at ×20 (Leica DM2000; Leica DFC450C; L.A.S V4.5; Leica Microsystems, Heidelberg, Germany). Each structure analyzed was explored on 3 sections per animal, positioned homogeneously in the rostro-caudal extension between animals using anatomical markers. Only microglial cells with a clearly visible soma were included in the study to minimize staining artefact. Iba1 staining was analyzed using the skeletal analysis plugin on Fiji as previously published [10, 11]. Microphotographs were pre-processed by converting to 8-bit and applying an FFT bandpass filter. The brightness/contrast of the microphotography was then adjusted to best visualize the soma and branches of microglia. Unsharp mask was then applied to increase the

contrast, and the despeckle function was used to remove pixels/noise. The threshold was then adjusted and closed, and the 'remove outliers' function was applied. A binarized image of microglia was then skeletonized. Microglial cell density, branch number per microglia, and branch length were analyzed.

RNA Sequencing and Quantitative RT-PCR

Total RNAs were extracted from freshly collected tissue stored at -80°C (spinal cord, medulla oblongata, and pons; sham, n = 4, and tsDCS, n = 3) using the Nucleospin RNA II kit (Macherey-Nagel, Düren, Germany, Cat. #740955) according to the manufacturer's protocol. RNA quality and quantity were evaluated using BioAnalyzer 2100 (Agilent Technologies, Santa Clara, CA, USA) with the RNA 6000 Nano Kit (Agilent Technologies, Cat. #5067-1511).

RNA sequencing libraries were constructed from 250 ng of total RNA using a modified TruSeq RNA Sample preparation kit protocol at the IGenSeq genomic sequencing platform (Institut du Cerveau, Paris, France). FASTQ files were processed by the Molecular and cellular tissue phenotyping using IDVseq (an in-house customized version of BEAVR [12]). Briefly, RNAseq FASTQ files were aligned to the mouse genome and counted using STAR v2.7.9a. The count file and sample file were loaded in an in-house R Shiny application, EYE DV seq (Phénotypage Cellulaire et Tissulaire, Institut de la Vision, Paris, France). Normalization and differential expression analysis values were computed with DESeq 2. The RNA-Seq raw fastq files were deposited in the European Nucleotide Archive (ENA) under the project accession number PRJEB102185.

Total RNA was reverse-transcribed into cDNA using a High-Capacity RNA to cDNA kit (Life Technologies, ref. 4,368,814) according to the manufacturer's instructions. cDNA was diluted in DNAse/RNAse-free water (Gibco) to a final concentration of 5 ng/µL. Real-time PCR was performed using the TaqMan Universal PCR Master Mix (Applied Biosystems, ref. 4,324,018) and an Applied Biosystem Quant Studio 5 instrument (Applied Biosystems). The delta-delta Ct method (ddCt) was used to analyze relative gene expression. *Gapdh* was used as a housekeeping gene. Primers were purchased from Applied Biosystems: *Egr2* (Mm00456650_m1), *Egr4* (Mm00842279_g1), *Fosb* (Mm00500401_m1), and *Gapdh* (Mm99999915_g1).

Statistics

Physiological variables, histological data, and RT-qPCR data were analyzed using Prism 10 (GraphPad, San Diego, California, United States). Normality of data distribution for ventilatory variables (f_R, V_T, $\dot{V}_E$), SpO_2, c-FOS immunolabelled cells, and quantitative data of microglia morphology was assessed with the Kolmogorov-Smirnov Normality test. Depending on whether the distribution was normal or not, data were expressed, respectively, as mean ± standard error of the

mean or median and interquartile range [Q1; Q3]. For physiological variables: 1. Within the sham or tsDCS mouse groups, we used a repeated measure ANOVA or mixed model followed by the Benjamini, Krieger, and Yekutieli multiple comparison test. 2. Between the sham and tsDCS mouse groups, we used an ANOVA two-way or mixed model followed by the Benjamini, Krieger, and Yekutieli multiple comparison test. For histological and RT-qPCR data, a comparison between sham and tsDCS mice was carried out using an unpaired t-test or a Mann-Whitney test. Differences were considered significant if $P < 0.05$. RNA-seq results were filtered for the significant gene using the *P*-value (<0.05). A Principal Component Analysis (PCA) was made to illustrate the distribution of the sample between the regions of the central nervous system analyzed.

Supplementary Results

Improvement of Spontaneous Ventilation of *Lep$^{ob/ob}$* Mice Modeling OHS by Repeated Cathodal Cervical tsDCS

At D0, *Lep$^{ob/ob}$* mice had the following respiratory variables: f_R, 242±42 respiratory cycles.min^{-1}; V_T, 5.45±0.95 µL.g^{-1}; $\dot{V}_E$, 1.369±0.24mL.g^{-1}.min^{-1}. Their SpO_2 was at 94.9% [93.3;95.7]. Prior to all procedures, mean f_R, V_T, and $\dot{V}_E$ were homogeneous between the tsDCS and sham group. On D0, tsDCS and sham mice had similar SpO_2 (Table S2).

In sham mice, repeated anesthesia led to a temporary increase in f_R from D0 at d8 and a decrease in V_T at d10 (Fig. 1C, 1D). SpO_2 of sham mice showed no significant difference throughout the 5 days during which mice were engaged in sham procedures (94.9% [92.8;96.0] at D0 and 94.5% [79.0;97.1] at D5). Their time mice spent with SpO_2 ≤95% was also unchanged (Table S2).

Stability of Body Weight and Heart Rate of *Lep$^{ob/ob}$* mice throughout the Repeated Cathodal Cervical tsDCS or Sham Procedures

In sham and tsDCS mice, the body weight remained unchanged from D1 to D5 compared to the values measured on D0: sham mice D1 0.1g [-0.6;0.6], D2 0.3g [0.2;0.8], D3 0.9g [0.3;1.4], D4 0.9g [0.3;1.1] and D5 1.0g [-0.1;2.0]; tsDCS mice D1 0.3g [-3.2;0.6], D2 0.5g [-0.4;1.2], D3 0.7g [-0.1;1.5], D4 0.8g [-0.5;1.2] and D5 0.2g [-0.9;1.4]; two-way ANOVA analysis (F=0.725, P =0.576). On each day of the protocol, data from sham and tsDCS mice did not differ; two-way ANOVA analysis (F=1.001, P =0.323).

Heart rate values did not change from D1 to D5 compared to the values measured on D0, for either sham or tsDCS mice: sham mice D1 2.6 bpm [-40.1;16.2], D2 -49.6bpm [-118.4;81.0], D3 -1.8bpm [-42.9;55.9], D4 -11.8bpm [-44.5;48.3] and D5 -17.1bpm [-47.1;47.8] ; tsDCS mice D1 -38.0bpm [-51.1;-20.1], D2 -61.1bpm [-95.1;-2.5], D3 -24.9 [-38.3;16.9], D4 -42.6 [-68.5;-17] and D5

-17.5 [-66.1;-8.5]; two-way ANOVA analysis (F=0,869, P =0.43). On each day of the protocol, data from sham and tsDCS mice did not differ; two-way ANOVA analysis (F=1.562, P =0.24).

Enhancement of Spontaneous Ventilation of WT OF1 Mice by Repeated Cathodal Cervical tsDCS

At D0, OF1 mice had the following respiratory variables: f_R, 198±8 respiratory cycles.min^{-1}; V_T 6.0±0.2 µL.g^{-1}; $\dot{V}_E$ 1.2±0.05 mL.g^{-1}.min^{-1}.

Repeated Cathodal Cervical tsDCS in *Lep$^{Ob/Ob}$* Mice Increased the Number of cFOS-positive Cells in Respiratory-related Structures of the Ventral Medulla Oblongata

In the medulla oblongata, the number of cFOS-positive cells was increased by cervical cathodal tsDCS in ventral structures of the respiratory network without any change at the dorsal level (Table S3).

tsDCS-induced Gene Expression Changes in the Cervical Spinal Cord, Medulla Oblongata, and Pons Revealed Neuroplasticity Phenomenon

Principal Component Analysis (PCA) plots illustrating the distribution of samples between the central nervous system analyzed regions (Fig. S1A) and between the two conditions (tsDCS vs sham) for each analyzed region (Fig. S1B, S1C, S1D) are shown.

RNAseq analysis focused on the cervical spinal cord, where tsDCS had been applied, as well as on the medulla oblongata and pons, *i.e.,* the brain regions that contain the respiratory network controlling the central respiratory drive, showed that the number of genes whose expression is modulated was limited (Table S4).

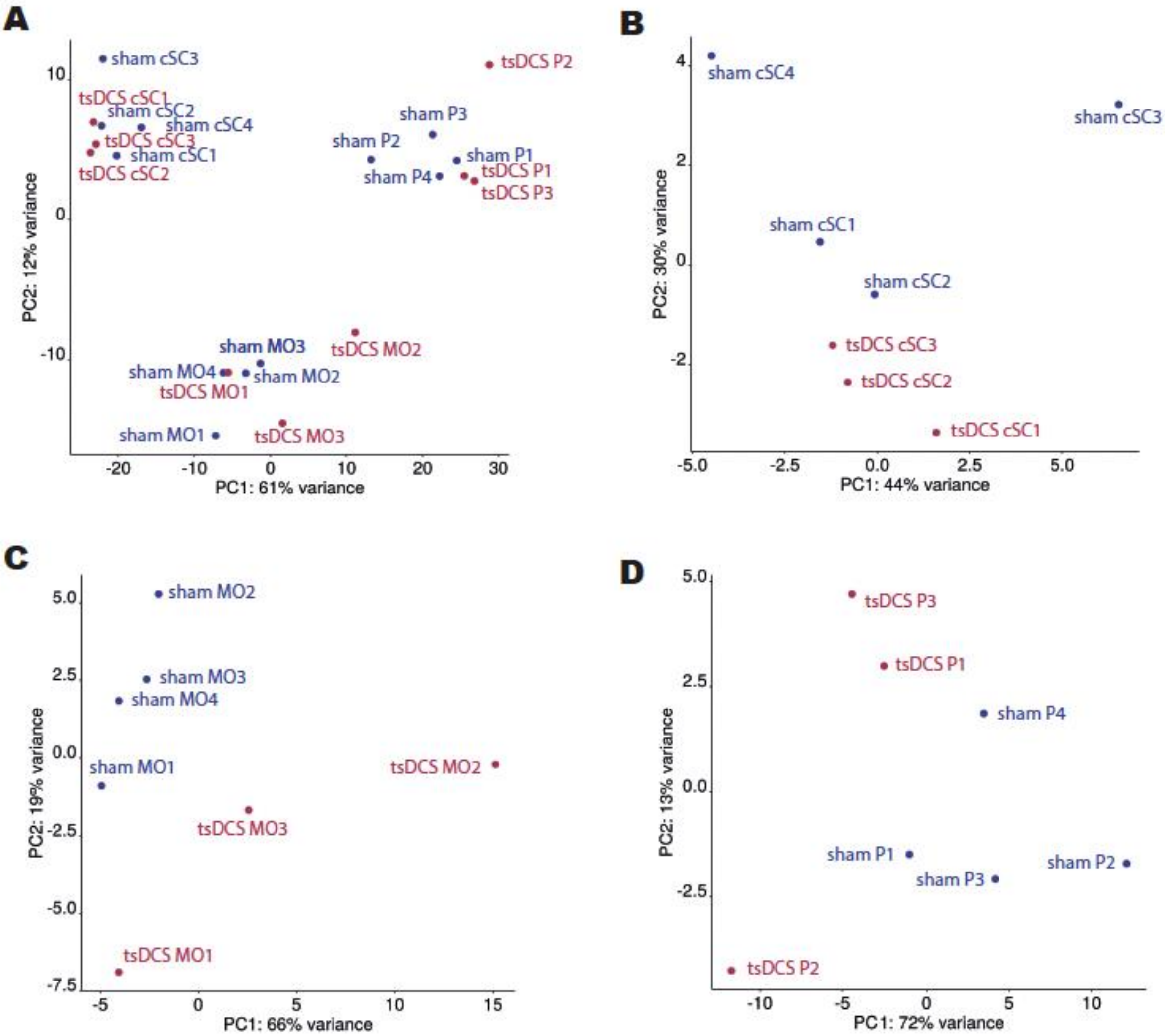

Fig. S1. Principal Component Analysis (PCA) Plot Showing the Distribution of Samples Across the Two Conditions: Cervical Cathodal tsDCS versus Sham. A, samples of the three central nervous system regions analyzed are represented: the cervical spinal cord (cSC), medulla oblongata (MO), and pons (P). **B**, samples of cSC. **C**, samples of the MO. **D**, samples of the P.

tsDCS did not lead to Changes in Microglia Morphology in Respiratory-related Structures with Increased Density of cFOS-positive Cells.

We did not observe any changes in microglial cell density or in the number and length of cell branches in any structure related to respiratory control that showed a change in cFOS distribution (Table S5).

References

[1] Tankersley C, Kleeberger S, Russ B, Schwartz A, Smith P. Modified control of breathing in genetically obese (ob/ob) mice. J Appl Physiol 1996, 81: 716–723.

[2] O'Donnell CP, Schaub CD, Haines AS, Berkowitz DE, Tankersley CG, Schwartz AR, *et al*. Leptin prevents respiratory depression in obesity. Am J Respir Crit Care Med 1999, 159: 1477–1484.

[3] Ahmed Z. Effects of cathodal trans-spinal direct current stimulation on lower urinary tract function in normal and spinal cord injury mice with overactive bladder. J Neural Eng 2017, 14: 056002.

[4] Paxinos G, Franklin KB. The mouse brain in stereotaxic coordinates, 2nd Edition. San Diego, CA: Academic Press, 2001.

[5] da Silva CA Jr, Picardo MCD, Del Negro CA. Ion channels in respiratory rhythm generation and sensorimotor integration. Neuron 2025, 113: 3530–3553.

[6] Delucenay-Clarke R, Niérat MC, Frugière A, Similowski T, Cayetanot F, Bodineau L. Direct current stimulation as a non-invasive therapeutic alternative for treating autonomic or non-autonomic neurological disorders affecting breathing. Clin Auton Res 2024, 34: 395–411.

[7] Dubreuil V, Ramanantsoa N, Trochet D, Vaubourg V, Amiel J, Gallego J, *et al*. A human mutation in Phox2b causes lack of CO_2 chemosensitivity, fatal central apnea, and specific loss of parafacial neurons. Proc Natl Acad Sci U S A 2008, 105(3): 1067–1072.

[8] Voituron N, Frugière A, Champagnat J, Bodineau L. Hypoxia-sensing properties of the newborn rat ventral medullary surface *in vitro*. J Physiol 2006, 577: 55–68.

[9] Shi Y, Stornetta RL, Stornetta DS, Onengut-Gumuscu S, Farber EA, Turner SD, *et al*. Neuromedin B expression defines the mouse retrotrapezoid nucleus. J Neurosci 2017, 37: 11744–11757.

[10] Green TRF, Murphy SM, Rowe RK. Comparisons of quantitative approaches for assessing microglial morphology reveal inconsistencies, ecological fallacy, and a need for standardization. Sci Rep 2022, 12: 18196.

[11] Young K, Morrison H. Quantifying microglia morphology from photomicrographs of immunohistochemistry prepared tissue using ImageJ. J Vis Exp 2018(136): e57648.

[12] Perampalam P, Dick FA. BEAVR: A browser-based tool for the exploration and visualization of RNA-seq data. BMC Bioinform 2020, 21: 221.
