# Supplementary material for: Noninvasive Trans-Spinal Direct Current Stimulation Improves Breathing in Mice Modeling an Obesity-Associated Central Hypoventilation Syndrome
Source: Neurosci Bull. 2026 Jul 24;42(10):2393–8. doi: 10.1007/s12264-026-01680-9 (PMC13642236; doi:10.1007/s12264-026-01680-9)
Supplement: Supplementary file 2 [file 12264_2026_1680_MOESM2_ESM.pdf]

Supplementary Materials

Table S1 Effects of Cervical Cathodal tsDCS on the Time Spent Under $SpO_2 \leq 95$ in *Lep^ob/ob^* mice

	% of time spent - $SpO_2 \leq 95$	
day	sham	tsDCS
D0	46.9 [25.6;56.0]	58.6 [18.6;87.3]
D1	35.3 [19.2;43.7]	64.4 [32,8;86.5] *$*
D2	18.4 [4.4;53.0]	6.7 [0.4;28.8] **
D3	44.6 [25.4;57.3]	10.8 [7.9;40.1] *
D4	59.4 [34.2;84.2]	24.9 [9.1;34.4] *;$
D5	39.4 [11.8;47.6]	7.2 [4.4;41.1] *

% of time spent with a $SpO_2 \leq 95$ is expressed as median [Q1; Q3]. Cohorts were $n = 5$ for tsDCS mice and $n = 5$ for sham mice. All comparisons were tested with two-way ANOVA analysis. *, intragroup comparisons ($F = 3.049$, $P<0.0175$) and $, intergroup comparisons ($F = 2.802$, $P <0.026$); * $P <0.05$, ** $P <0.01$, $ $P <0.05$

Table S2 Effects of 5 consecutive days of Cervical Cathodal tsDCS on Breathing Variables in WT OF1 Mice

	f_R (cycle.min^{-1})		V_T (µL.g^{-1})		$\dot{V}_E$ (mL.g^{-1}.min^{-1})	
day	sham	tsDCS	sham	tsDCS	sham	tsDCS
D0	216 [196;232]	166 [158;217]	5.91 [5.45;6.22]	5.60 [5.27;6.83]	1.31 [1.16;1.40]	1.05 [0.86;1.34]
D1	200 [168;234]	230 [207;259] **	6.52 [4.81;7.29]	5.67 [5.48;7.33]	1.24 [1.01;1.66]	1.47 [1.32;1.63] *
D2	204 [181;233]	294 [251;300] ****; $$$$	6.04 [4.95;6.66]	6.23 [5.22;6.87]	1.27 [1.00;1.36]	1.69 [1.49;1.83] **** ; $$
D3	201 [180;252]	280 [272;310] **** ; $$$$	6.06 [5.67;7.60]	6.26 [5.08;7.17]	1.26 [1.12;1.62]	1.99 [1.51;2.08] **** ; $$$
D4	208 [189;231]	303 [296;335] **** ; $$$$	7.03 [5.62;7.73] *	6.39 [5.36;6.87]	1.45 [1.15;1.63]	1.94 [1.60;2.26] **** ; $$$
D5	170 [162;200] *	284 [274;318] **** ; $$$$	6.60 [6.20;8.24] **	6.63 [5.92;7.39]	1.29 [1.14;1.41]	1.85 [1.65;2.18] **** ; $$$$

f_R, V_T and $\dot{V}_E$ are expressed as median [Q1; Q3]. Cohorts were $n = 11$ for tsDCS mice and $n = 11$ for sham mice. All comparisons were tested with two-way ANOVA analysis or mixed model followed by the Benjamini, Krieger, and Yekutieli multiple comparison test. *, intragroup comparisons (f_R $F = 10{,}85$, $P < 0.0001$; V_T $F = 2{,}765$, $P = 0.0221$; $\dot{V}_E$ $F = 8.024$, $P < 0.0001$) and $, intergroup comparisons (f_R $F = 12.7$, $P < 0.0001$; $\dot{V}_E$ $F = 4.725$, $P = 0.0006$); * $P < 0.05$, ** $P < 0.01$, *** $P < 0.001$, **** $P < 0.0001$, $ $P < 0.05$, $$ $P < 0.01$, $$$ $P < 0.001$, $$$$ $P < 0.0001$

Table S3 c-FOS-positive Number of Cells in Respiratory-related Ponto-medullary Structures in Sham and tsDCS *Lep*$^{Ob/Ob}$ Mice

	Sham	tsDCS
Medulla Oblongata		
12N	0.38 [0.05; 0.68]	0.14 [0.09; 0.35]
10N	4.46 [2.84; 5.45]	2.38 [1.12; 5.57]
Amb	5.73 [4.48; 7.10]	12.5 [8.99; 14.3] $^{\$\$}$
c/mNTS	30.9 [25.3; 48.0]	25.2 [24.5; 46.8]
pFRG	1.24 [0.49; 1.93]	2.50 [1.16; 4.27]
ROb	7.76 [4.52; 9.45]	12.2 [11.6; 13.5] $^{\$\$}$
RPa	5.79 [4.55; 7.65]	10.4 [8.45; 12.6] $^{\$\$}$
RTN	4.0 [2.97; 5.17]	8.95 [4.79; 11.3] $^{\$}$
VLM	12.8 [5.11; 21.0]	23.2 [19.2; 31.5] $^{\$}$
Pons		
KF	1.57 [1.04; 2.90]	3.17 [1.43; 6.16]
LC	2.4 [1.51; 8.98]	7.81 [5.40; 11.4]
lPB	11.2 [3.29; 23.7]	25.4 [12.5; 33.9]
mPB	4.35 [2.88; 7.03]	7.77 [4.68; 14.6]

Values are expressed as median [Q1; Q3] number of mean c-FOS-positive cells per section. Cohorts were n = 6 for sham and n = 6 for tsDCS mice. Unpaired t-test or a Mann-Whitney test; $^{\$}$ indicates a significant difference between sham and cervical cathodal tsDCS *Lep*$^{Ob/Ob}$ mice. $^{\$}$ $P < 0.05$, $^{\$\$}$ $P < 0.01$.

10N, dorsal motor nucleus of the vagus; 12N, hypoglossal nucleus; Amb, ambiguus nucleus; c/mNTS, commissural and median parts of the solitary tract nucleus; KF, Kölliker fuse nucleus; LC, *locus coeruleus*; lPB, lateral part of the parabrachial nucleus; mPB, medial part of the parabrachial nucleus; pFRG, parafacial respiratory group nucleus; ROb, nucleus of the raphe *obscurus*; RPa, nucleus of the raphe *pallidus*; RTN, retrotrapezoid nucleus; VLM, ventrolateral reticular nucleus of the medulla.

Table S4. Regulated Genes (Cervical Cathodal tsDCS versus Sham) Ranked by Alphabetic Order

Gene ID	Gene name	Log2FC	*P*adj
**Cervical Spinal Cord**			
1700016P03Rik	*RIKEN cDNA 1700016P03 gene*	1.7	3.6-04
Ch25h	*cholesterol 25-hydroxylase*	1.7	1.7-02
Egr2 *	*early growth response 2*	1.6	4.4-03
Egr4 *	*early growth response 4*	2.5	1.3-15
Fosb	*FBJ osteosarcoma oncogene B*	1.7	4.1-03
Gh *	*growth hormone*	-4.6	1.2-04
Icam1 * #	*intercellular adhesion molecule 1*	2.1	5.8-27
Lcn2 * #	*lipocalin 2*	1.7	1.5-03
Serpine1	*serine (or cysteine) peptidase inhibitor, clade E, member 1*	1.9	1.5-09
Zfp366	*zinc finger protein 366*	-1.8	6.6-05
**Medulla Oblongata**			
Car8 *	*carbonic anhydrase 8*	3.1	4.8-02
Cbln3 *	*cerebellin 3 precursor protein*	4.9	3.2-03
Cnpy1	*canopy FGF signaling regulator 1*	5.8	2.1-03
Crtam #	*cytotoxic and regulatory T cell molecule*	3.3	1.8-02
En2 *	*engrailed 2*	2.5	3.4-02
Eomes *	*eomesodermin*	4.4	2.4-02
Fat2	*FAT atypical cadherin 2*	4.4	5.1-03
Gabra6 *	*gamma-aminobutyric acid type A receptor subunit alpha 6*	5.8	2.1-03
Gabrd *	*gamma-aminobutyric acid (GABA) A receptor, subunit delta*	3.2	2.4-02
Gh *	*growth hormone*	-5.1	1.7-04
Hes3 *	*hes family bHLH transcription factor 3*	3.2	2.4-02
Icam1 * #	*intercellular adhesion molecule 1*	2.0	6.8-23
Il16 #	*interleukin 16*	3.3	3.5-02
Il20rb #	*interleukin 20 receptor beta*	3.9	2.6-03
Lcn2 * #	*lipocalin 2*	2.3	5.7-13
Ltbp2	*latent transforming growth factor beta binding protein 2*	4.5	3.1-03
Mybpc3	*myosin binding protein C, cardiac*	3.2	2.6-02
Neurod1 *	*neurogenic differentiation 1*	2.8	4.2-02
Pcp2	*Purkinje cell protein 2*	4.2	5.6-03
Ppp1r17	*protein phosphatase 1, regulatory subunit 17*	3.8	1.7-02
Zfp366	*zinc finger protein 366*	-2.2	1.4-27
**Pons**			
4933429O19Rik	*RIKEN cDNA 4933429O19 gene*	2.0	1.5-02
Ch25h	*cholesterol 25-hydroxylase*	1.8	1.3-02
Cldn1	*claudin 1*	1.8	4.5-13
Clic6 *	*chloride intracellular channel 6*	2.0	4.1-02
Gh *	*growth hormone*	-6.5	6.0-04
Gm13944	*predicted gene 13944*	2.4	1.0-02
Gm14033	*predicted gene 14033*	1.5	7.5-03
Icam1 * #	*intercellular adhesion molecule 1*	1.7	2.7-03
Krt18 *	*keratin 18*	1.9	2.1-07
Krt8	*keratin 8*	1.8	4.4-04
Lcn2 * #	*lipocalin 2*	2.3	1.7-03
Ldc1	*leucine decarboxylase 1*	2.1	3.3-02
Nlrp10 #	*NLR family, pyrin domain containing 10*	2.0	3.3-02
Oca2 *	*oculocutaneous albinism II*	1.7	2.1-02
Prr32	*proline rich 32*	2.2	9.0-09
Tc2n	*tandem C2 domains, nuclear*	1.6	1.4-02
Tmprss11a	*transmembrane protease, serine 11a*	2.4	9.6-03
Wdr86	*WD repeat domain 86*	2.0	1.2-08
Zfp366	*zinc finger protein 366*	-2.4	5.2-05

*P*adj, adjusted *P* -value

*, genes listed in the Gene Ontonlogy database as involved in neuronal transmission (synapse assembly or reinforcement, development or maturation of neuronal compartments, …), neuronal differentiation or regionalization of central nervous tissue, glial cell function or differentiation; #, genes listed in the Gene Ontonlogy database as involved in inflammatory processes or, more broadly, immunological processes.

Table S5. Morphological Characteristics of Microglial Cells in Sham and tsDCS $Lep^{Ob/Ob}$ Mice

	Mean number of microglia/structure		Mean microglial branch length (µm)		Mean number of microglial branch/microglia	
	Sham	tsDCS	Sham	tsDCS	Sham	tsDCS
Amb	7.0 [5.9;8.5]	5.3 [4.0;6.9]	7.8 [5.8;9.0]	7.5 [6.8;8.3]	23.3 [17.3;36.0]	27.0 [22.6;30.0]
ROb	8.6 [8.0;12.5]	8.3 [7.4;10.7]	8.1 [5.8;10.3]	7.9 [6.1;8.1]	15.5 [11.7;19.3]	19.3 [15.5;22.3]
RPa	8.8 [7.4;9.9]	10.1 [5.9;13.4]	7.8 [6.7;9.3]	7.2 [6.8;7.7]	12.9 [11.0;17.3]	18.5 [15.11 ;23.6]
RTN	8.5 [8.0;9.6]	8.3 [6.2;11.9]	7.8 [6.9;8.4]	7.8 [7.1 ;8.4]	9.5 [14.1 ;18.0]	11.1 [10.6 ;16.3]
VLM	5.8 [4.9;6.2]	6.0 [5.1;6.3]	7.1 [6.3;8.0]	7.0 [6.4;7.4]	14.1 [9.5;18.0]	11.2 [10.6;16.2]

Values are expressed as median [Q1; Q3]. Cohorts were n = 4 for tsDCS mice and n = 4 for sham mice. Comparisons were tested with Mann-Whitney, no significant difference were observed.

Amb, ambiguus nucleus; ROb, nucleus of the raphe *obscurus*; RPa, nucleus of the raphe *pallidus*; RTN, retrotrapezoid nucleus; VLM, ventrolateral reticular nucleus of the medulla.
